# Deguelin Potentiates Apoptotic Activity of an EGFR Tyrosine Kinase Inhibitor (AG1478) in *PIK3CA*-Mutated Head and Neck Squamous Cell Carcinoma

**DOI:** 10.3390/ijms18020262

**Published:** 2017-01-26

**Authors:** Yuh Baba, Toyonobu Maeda, Atsuko Suzuki, Satoshi Takada, Masato Fujii, Yasumasa Kato

**Affiliations:** 1 Department of General Clinical Medicine, Ohu University School of Dentistry, 31-1, Mitsumido, Tomita-machi, Koriyama City, Fukushima 963-8611, Japan; 2 Department of Oral Function and Molecular Biology, Ohu University School of Dentistry, 31-1, Mitsumido, Tomita-machi, Koriyama City, Fukushima 963-8611, Japan; t-maeda@den.ohu-u.ac.jp (T.M.); at-suzuki@den.ohu-u.ac.jp (A.S.); 3 Department of Oral and Maxillofacial Surgery, Ohu University School of Dentistry, 31-1, Mitsumido, Tomita-machi, Koriyama City, Fukushima 963-8611, Japan; s-takada@den.ohu-u.ac.jp; 4 National Institute of Sensory Organs, National Tokyo Medical Center, 2-5-1, Higashigaoka, Meguro, Tokyo 152-8902, Japan; fujiimasato@kankakuki.go.jp

**Keywords:** deguelin, epidermal growth factor receptor (EGFR), AKT, *PIK3CA*, head and neck squamous cell carcinoma (HNSCC)

## Abstract

Head and neck squamous cell carcinoma (HNSCC) is known to be intrinsically resistant to inhibitors for epidermal growth factor receptor (EGFR). Until now, clinical outcomes for HNSCC using EGFR inhibitors as single agents have yielded disappointing results. Here, we aimed to study whether combinatorial treatment using AG1478 (EGFR tyrosine kinase inhibitor) and deguelin, which is a rotenoid isolated from the African plant *Mundulea sericea*, could enhance the anti-tumor effects of AG1478 in HNSCC. For Ca9-22 cells with *EGFR*, *KRAS*, and *PIK3CA* wild types, AG1478 alone suppressed both phosphorylated levels of ERK and AKT and induced apoptosis. On the contrary, for HSC-4 cells with *EGFR* and *KRAS* wild types, and a *PIK3CA* mutant, AG1478 alone did not suppress the phosphorylated level of AKT nor induce apoptosis, while it suppressed ERK phosphorylation. Forced expression of constitutively active *PIK3CA* (G1633A mutation) significantly reduced the apoptotic effect of AG1478 on the *PIK3CA* wild-type Ca9-22 cells. When HSC-4 cells with the *PIK3CA* G1633A mutation were treated with a combination of AG1478 and deguelin, combination effects on apoptosis induction were observed through the inhibition of the AKT pathway. These results suggest that the combination of EGFR tyrosine kinase inhibitor with deguelin is a potential therapeutic approach to treat *PIK3CA*-mutated HNSCC.

## 1. Introduction

Epidermal growth factor receptor (EGFR) is a receptor tyrosine kinase that activates intracellular signaling pathways, including the phosphatidylinositol 3-kinase (PI3K)/AKT pathway (survival signal) and the ERK pathway (proliferation signal), and is abundantly expressed in the majority of epithelial malignancies, including head and neck squamous cell carcinoma (HNSCC) [1]. Because the elevated expression of EGFR in HNSCC correlates with poor prognosis, EGFR signaling has been thought to be the most important target in anticancer treatment strategies [2]. Therefore, the use of EGFR-targeting drugs, such anti-EGFR monoclonal antibody (cetuximab) and EGFR tyrosine kinase inhibitors (TKIs) (gefitinib and erlotinib), have been expected to be an applicable strategy for HNSCC therapy.

Somatic mutations in the tyrosine kinases domain of the *EGFR* gene (in-frame deletion in exon 19 and missense mutations, such as L858R, G719X, and L861Q) are the increased binding activity of EGFR TKIs at the ATP binding site of EGFR tyrosine kinase and are deeply associated with increased sensitivity to EGFR TKIs [3]. Activating mutations of the EGFR are frequently found in the EGFR TKI responder in non-small cell lung carcinoma (NSCLC) patients, the majority of which are “never smoker” Asian women [4]. These mutant EGFRs selectively activate the signal transduction and activator of transcription (STAT) and AKT signaling pathways, and the inhibition of those signals by EGFR inhibitors could contribute to the efficacy of a drug used to treat NSCLC [5]. On the other hand, a resistance mutation has emerged in EGFR. The T790M mutation increases tyrosine kinase affinity for ATP and, consequently, reduces the competitive binding of the EGFR TKIs to tyrosine kinase [6]. Previously, we could find neither sensitive nor resistance mutations in HNSCC patients, which is different from those found in lung cancers [7].

Early clinical studies with EGFR TKIs as single agents have turned out to be disappointing; that is, the respective overall response rates for gefitinib and erlotinib were 11% [8] and 4% [9] in patients with recurrent and/or metastatic HNSCC. Although it has been reported that an EGFR variant is a possible reason for resistance to EGFR targeting in HNSCC [10], the exact mechanisms of EGFR TKI resistance are incompletely understood. A promising solution to improve the clinical response rate may be both the identification of biomarkers to predict the EGFR TKI’s efficacy and the combination of EGFR TKIs with other treatment modalities for patients who are predicted as non-responders.

Although the *KRAS* mutation, which resulted in EGFR-independent ERK activation, was suggested to be a potential biomarker for predicting the efficacy of EGFR TKI in lung cancer [11], it was rare in HNSCC [12]. On the contrary, activation of the PTEN/PIK3CA/AKT pathway by the *PIK3CA* mutations has been reported in HNSCC [13,14]. Although it seemed that EGFR-independent AKT activation by the mutation possibly occurs and contributes to the resistance to EGFR TKI, it has also been reported that the loss of PTEN expression was not associated with resistance to cetuximab in HNSCC [15]. Thus, it is still controversial whether mutation of the PTEN/PIK3CA/AKT pathway is associated with the sensitivity of EGFR inhibitors.

Deguelin, which is a rotenoid isolated from the African plant *Mundulea sericea* (Leguminosae), is a potent chemopreventive agent for some kinds of cancers, e.g., aberrant crypt foci in colons [16], skin papilloma [17,18], lung tumor [19], and mammary grand adenocarcinoma [18]. In recent years, the molecular mechanisms of deguelin’s function have been uncovered, such as the inhibition of AKT signaling, disruption of the survivin-heat shock protein 90 complex, and inductions of ubiquitin-mediated degradation of cyclin-dependent kinase 4 and autophagy-mediated apoptosis through the ceramide-AMP-ctivated protein kinase-Ulkl axis [20]. Furthermore, we recently reported that deguelin induced apoptosis by targeting both the EGFR-AKT and IGF1R-AKT pathways in HNSCC cell lines [21] and suggested that AKT signaling underlies EGFR inhibitor resistance in HNSCC [7].

In this study, we analyzed whether the possible biomarker of the response to an EGFR TKI, AG1478, could be a *PIK3CA* mutation. Next, we investigated the anti-tumor effects of the combination of AG1478 and deguelin in vitro using an HNSCC cell line that was not sensitive to AG1478.

## 2. Results

### 2.1. Head and Neck Squamous Cell Carcinoma (HNSCC) Cell Lines after AG1478 Treatment

#### 2.1.1. AG1478 Suppressed the Phosphorylation of EGFR in a Dose-Dependent Manner in HSC4 Cells

**Figure 1:**
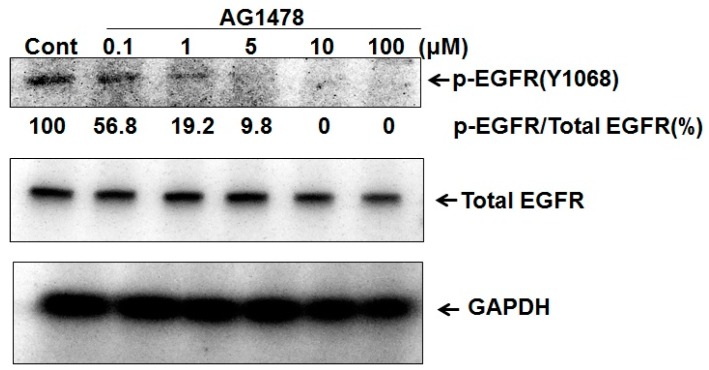
AG1478 suppressed p-EGFR level in dose-dependent manner in HSC-4 cells. Subconfluent HSC-4 cells were incubated for 24 h in serum-free medium. After the starvation, cells were treated with variable concentrations of AG1478 for a 1 h incubation. The AG1478-treated HSC-4 cells were further incubated for 15 min with 10 ng/mL of epidermal growth factor (EGF). Whole-cell extracts were analyzed by Western blot using antibodies against p-EGFR and EGF receptor (EGFR).

In this study, we used two representative HNSCC cell lines with different *PIK3A* gene statuses (Table 1). At first, we tested the dose-dependency of AG1478 in the down-regulation of EGFR phosphorylation. Figure 1 clearly shows that AG1478 inhibited the phosphorylated level to 56.8% at 0.1 μM, and completely at 10 μM or more.

#### 2.1.2. AG1478 Did Not Induce Apoptosis in HSC-4 (Mutant PIK3CA), but Induced Apoptosis in Ca9-22 Cells (WT PIK3CA)

**Figure 2:**
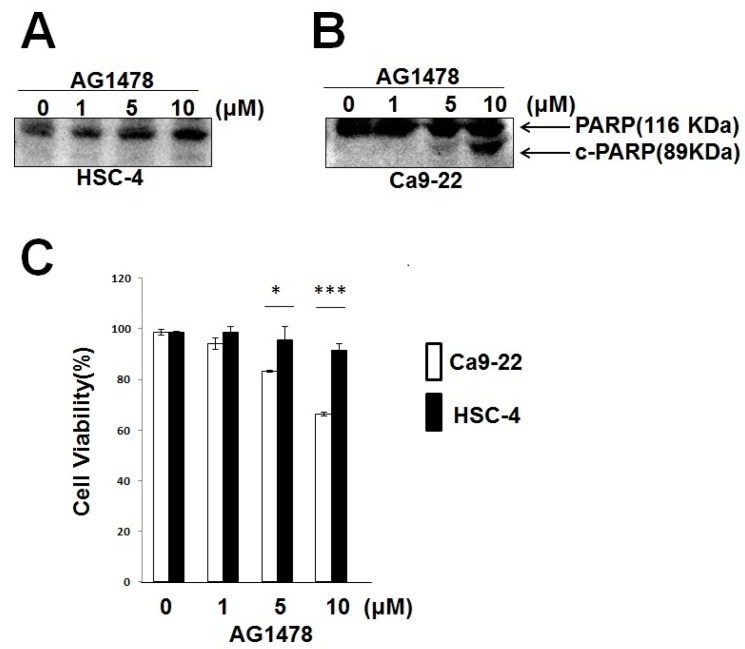
AG1478 did not induce apoptosis in HSC-4, but induced apoptosis in Ca9-22 cells. Subconfluent cultures were incubated with AG1478 for 72 h in the presence of 10% FBS without serum starvation. Western blot was performed to detect the cleaved-PARP (c-PARP) after AG1478 treatment in HSC-4 (**A**) and Ca9-22 cells (**B**); (**C**) Trypan-blue exclusion assay was performed to measure cell viability of HSC-4 cells for 72 h after AG1478 treatment. Cell viability (%) was calculated by viable cell number/total cell number (viable and dead cells) at each concentration. The values were expressed as the means ± SE (a representative of at least three independent experiments). * *p* < 0.05, *** *p* < 0.001.

Because the activation of EGFR leads to the activation of intracellular signaling pathways, including the PI3K/AKT pathway (survival signal), the inhibition of EGFR activation by AG1478 may induce apoptosis, dependent on the *PIK3CA* gene status. Therefore, we examined the effects of AG1478 on cancer cells with different *PIK3CA* gene statuses. Expectedly, HSC-4 cells with a mutant *PIK3CA* gene did not undergo apoptosis by AG1478 treatment, which was enough of a concentration for the inhibition of EGFR phosphorylation (Figure 2A). However, for Ca9-22 cells with the WT *PIK3CA* gene, AG1478 induced cleavage of PARP (c-PARP) as a consequence of apoptosis, apparently at 5 μM, and clearly at 10 μM for 72 h (Figure 2B). This difference was also shown by a cell viability assay. Reduction of cell viability by AG1478 was shown at a much higher rate in Ca9-22 cells than in HSC-4 cells (Figure 2C). These data suggested that the apoptotic effect of AG1478 was deeply associated with the *PIK3CA* gene status.

#### 2.1.3. While AG1478 Suppressed the Expression of Both Phosphorylated ERK and AKT in Ca9-22 Cells (WT PIK3CA), AG1478 Suppressed the Expression of Phosphorylated ERK and Did Not Significantly Suppress the Expression of Phosphorylated AKT in HSC-4 Cells (Mutant PIK3CA)

**Figure 3:**
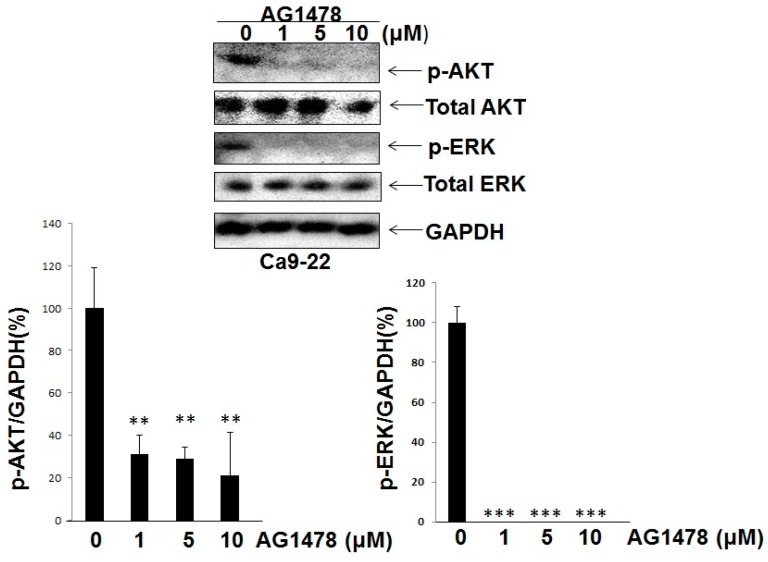
AG1478 suppressed p-ERK and p-AKT levels in Ca9-22 cells. Subconfluent Ca9-22 cells in DMEM + 10% fetal bovine serum (FBS)were treated with or without AG1478 for 2 h in the presence of 10% FBS without serum starvation. Whole-cell extracts were analyzed by Western blot using antibodies against p-AKT and p-ERK. Intensities of Western blot signals were quantified by densitometric analyses. The values were expressed as the means ± SE (a representative of at least three independent experiments). ** *p* < 0.01, *** *p* < 0.001.

**Figure 4:**
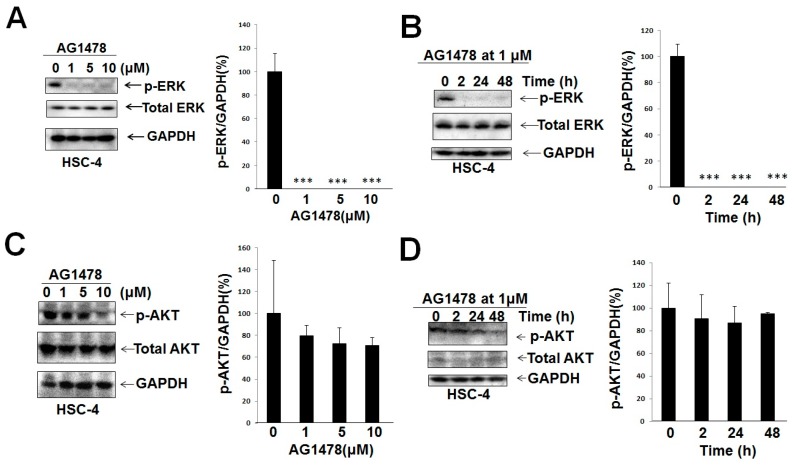
AG1478 did not significantly suppress p-AKT level but suppressed p-ERK level in HSC-4 cells. Subconfluent HSC-4 cells in DMEM + 10% FBS were treated with or without AG1478 for 2 h in the presence of 10% FBS without serum starvation. Western blot was performed to detect p-ERK (**A**,**B**) and p-AKT (**C**,**D**) levels in HSC-4. Intensities of Western blot signals were quantified by densitometric analyses. The values were expressed as the means ± SE (a representative of at least three independent experiments). *** *p* < 0.001.

Next, we compared the effects of AG1478 on survival signaling through AKT and proliferative signaling through ERK on Ca9-22 with the case of HSC-4 cells. Although AG1478 suppressed both phosphorylation of ERK and AKT in Ca9-22 cells (Figure 3), inhibition of ERK phosphorylation was only seen in HSC-4 cells (Figure 4).

#### 2.1.4. The Sensitivity of HNSCC Cells to AG1478 Depended on *PIK3CA* Gene Status

**Figure 5:**
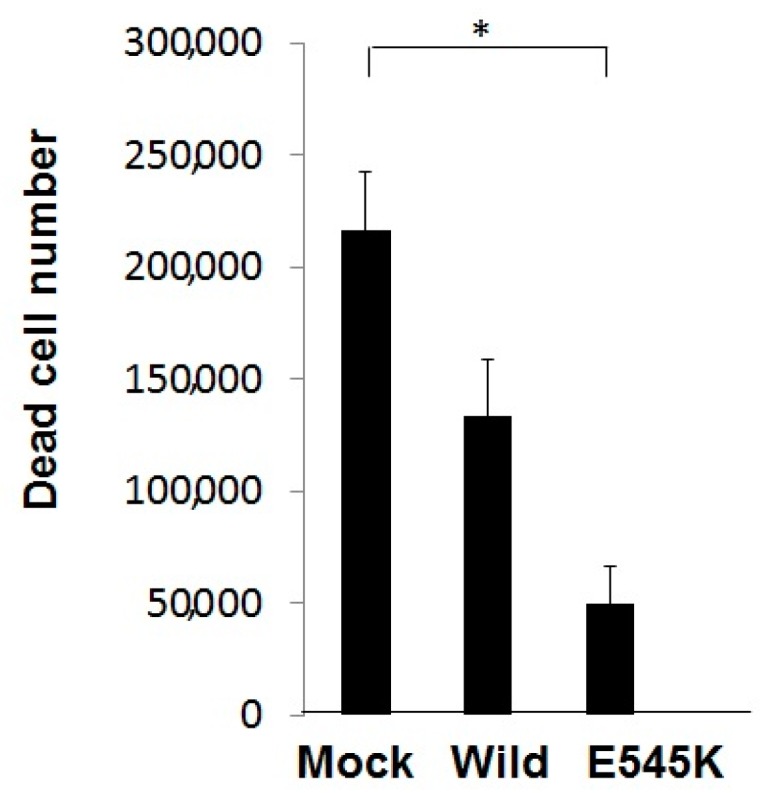
The sensitivity of HNSCC cells to AG1478 depends on PIK3CA gene status. Ca9-22 cells were transfected with the expression vector as follows: Empty vector (Mock), wild-type PIK3CA (Wild), or PIK3CA mutant (E545K). Each transfectant was treated with AG1478 (10 μM) for 72 h, and analyzed for cell viability according to the Trypan blue exclusion method. The values were expressed as the means ± SE (a representative of at least three independent experiments). * *p* < 0.05.

The above-mentioned results revealed the possible relationship between PI3K mutation and AG1478 efficacies. To obtain more direct evidence for this association, we introduced an expression vector of the mutant *PIK3CA* gene (G1633A in exon 9, E545K) in Ca9-22 cells, having the wild-type *PIK3CA* gene dominantly express mutant PI3K (constitutively active). Expectedly, enforced expression of the mutant *PIK3CA* gene prevented the cytotoxic effect of AG1478 on Ca9-22 cells, as compared with WT *PIK3CA* gene transfected cells and parental Ca9-22 cells (Figure 5). Thus, our results indicated that the sensitivity of HNSCC cells to AG1478 depends on the *PIK3CA* gene status.

### 2.2. Deguelin Suppressed Phosphorylation Level of AKT in Dose- and Time-Dependent Manner in HSC-4 Cells

**Figure 6:**
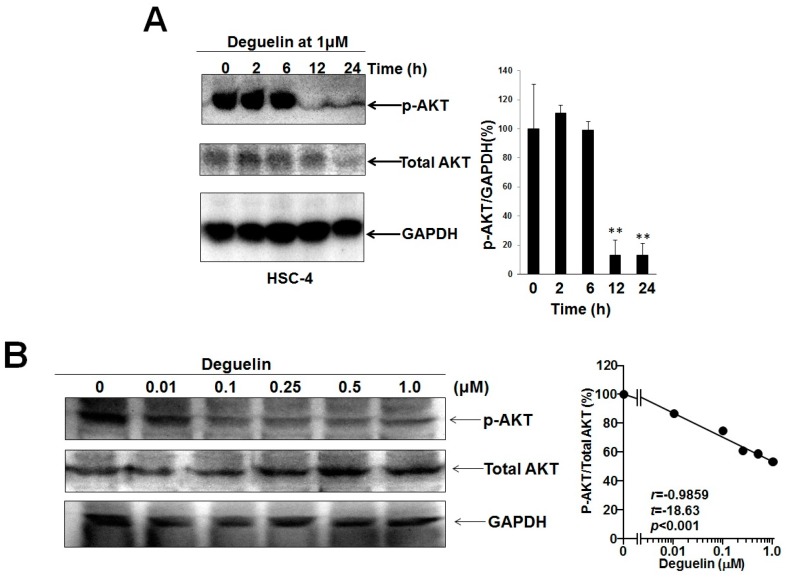
Deguelin suppressed p-AKT level in a time- and dose-dependent manner in HSC-4 cells. Subconfluent HSC-4 cells in DMEM + 10% FBS were treated with or without deguelin in the presence of 10% FBS without serum starvation: Time course study at 1 μM (**A**), dose-dependency during 12 h incubation (**B**). Western blot was performed to detect p-AKT levels in HSC-4 cells. Intensities of Western blot signals were quantified by densitometric analyses. The values were expressed as the means ± SE (a representative of at least three independent experiments). ** *p* < 0.01.

We next examined the inhibitory effect of deguelin on phosphorylation levels of AKT in HSC-4 cells. Figure 6 shows that the inhibitory effect of deguelin on AKT phosphorylation in HSC-4 cells was dose- and time-dependent in HSC-4 cells (Figure 6). Inhibition of AKT phosphorylation was obvious with 1 μM of deguelin treatment for 12 h, and this inhibition was prolonged to at least 24 h (Figure 6A). The inhibitory effect of deguelin for 12 h on AKT phosphorylation was significantly dose-dependent (Figure 6B). The best-fitting line on the semi-log plot of Figure 6B indicated a strong exponential relation between the deguelin concentration and AKT phosphorylation.

### 2.3. The Combination of AG1478 and Deguelin in HSC-4 Cells

Finally, we tested the effects of the combination of AG1478 and deguelin in HSC-4 cells.

#### 2.3.1. Combination of AG1478 and Deguelin Was Stronger Than AG1478 Alone with Regard to the Effect on the Active Form of AKT, and the Combination Was Stronger than Deguelin Alone with Regard to the Effect on the Active Form of ERK in HSC-4 Cells

**Figure 7:**
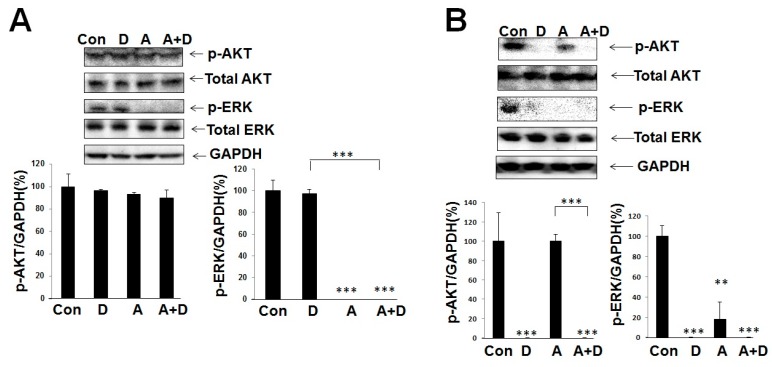
Deguelin and AG1478 show a combination effect on p-AKT and p-ERK levels. HSC-4 cells were incubated in the presence of deguelin (1 μM) and/or AG1478 (1 μM) for 2 h (**A**) and for 12 h (**B**). Western blot was performed to detect p-AKT and p-ERK levels in HSC-4 cells. Intensities of Western blot signals were quantified by densitometric analyses. The values were expressed as the means ± SE (a representative of at least three independent experiments). Con, control; D, deguelin; A, AG1478; A + D, AG1478 and Deguelin. ** *p* < 0.01, *** *p* < 0.001.

To test whether deguelin has activity for the sensitization of AG1478, we treated cells with AG1478 in the presence of deguelin to see the active forms of AKT and ERK. The 2 h combination treatment of deguelin and AG1478 significantly reduced phosphorylated levels of ERK, but not AKT, compared to the control or deguelin alone (Figure 7A). Longer treatment (12 h) with the combination significantly reduced AKT phosphorylation relative to the control or AG1478 alone (Figure 7B).

#### 2.3.2. The Combination of Deguelin and AG1478 Significantly Induced Apoptosis Relative to the Control or Alone in HSC-4 Cells

**Figure 8:**
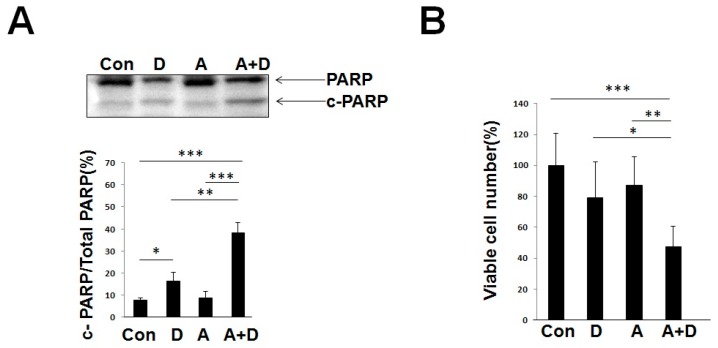
Deguelin and AG1478 show combination effects on the viability of HSC-4 cells. (**A**) HSC-4 cells were incubated in the presence of deguelin (1 μM) and/or AG1478 (1 μM) for 24 h. Western blot was performed to detect the cleaved-PARP (c-PARP) in HSC-4. Intensities of Western blot signals were quantified by densitometric analyses (% of c-PARP against total PARP (sum of PARP and c-PARP)); (**B**) After HSC-4 cells were incubated in the presence of deguelin (0.1 μM) and/or AG1478 (1 μM) for 24 h, trypan-blue exclusion assay was performed to measure cell viability of HSC-4 cells. The values were expressed as the means ± SE (a representative of at least three independent experiments). Con, control; D, deguelin; A, AG1478; A + D, AG1478 and Deguelin. * *p* < 0.05, ** *p* < 0.01, *** *p* < 0.001.

Finally, we determined the combination effect of deguelin and AG1478 on the apoptosis of HSC-4 cells. As shown in Figure 8A, deguelin (1 μM) alone significantly increased c-PARP levels to 2.1-fold higher than the control, while AG1478 (1 μM) alone slightly increased c-PARP levels to 1.1-fold higher than the control. When the cells were treated with both drugs, the c-PARP level increased up to 5.0-fold compared to the control, 4.4-fold compared to AG1478 alone, and 2.4-fold compared to deguelin alone, respectively. Furthermore, as shown in Figure 8B, AG1478 at 1 μM alone did not significantly reduce the proliferation of HSC-4 cells (see Figure 2C); however, the combination of deguelin at 0.1 μM and AG1478 at 1 μM significantly reduced the viable cell number of HSC-4 cells relative to the control or alone. Even a low dose of deguelin (0.1 μM) can significantly enhance the anti-tumor efficacy of AG1478.

## 3. Discussion

Overexpression of EGFR is observed in a majority of studied HNSCC cases [1]. Thus, the use of EGFR inhibitors, such as cetuximab, gefitinib, and erlotinib, has been expected to be an applicable strategy for HNSCC therapy. However, their monotherapy yielded low response rates in clinical trials [8,9]. A promising solution to improve the clinical response rate may be through the combination of EGFR inhibitors with other treatment modalities. Here, we demonstrated that deguelin has a combination effect with AG1478 in *PIK3CA* mutant HNSCC cells. Previously, deguelin alone was reported to induce apoptosis via AKT signaling inhibition, autophagy, and CDK4/Survivin degradation at high doses (50 or 100 μM) [20] and to suppress invasive ability at a low dose (0.1 μM) in HNSCC cells [23]. Although deguelin was reported to sensitize HNSCC cells to 5-fluorouracil (FU) [20], to the best of our knowledge, this is the first report that deguelin has a combination effect with an EGFR TKI for induction of apoptosis in HNSCC cells.

Previously, EGFR inhibitor–based therapy has been developed in combination with other inhibitory drugs targeting PI3K signaling. Rebucci et al. [24] demonstrated that the combination of cetuximab (EGFR antibody) with a PI3K inhibitor could be a good therapeutic option in *PIK3CA*-mutated HNSCC. Furthermore, D′Amato et al. [25] reported that the dual PI3K/mTOR inhibitor, PKI-587, enhances sensitivity to cetuximab in Detroit562 cells (HNSCC) having the *PIK3CA* mutant. On the other, in some cases, it has been reported that the combination of erlotinib (EGFR TKI) and everolimus (mTOR inhibitor) did not show significant benefits in unselected patients with platinum-resistant metastatic HNSCC [26], and that six out of 12 patients administered erlotinib and temsirolimus (mTOR inhibitor) withdrew within six weeks due to toxicity or death due to poor tolerance, prompting early closure of the trial [27]. Thus, combination therapy with an EGFR inhibitor and PI3K pathway inhibitor still seeks to provide better benefits for not only clinical efficacy, but also low side effects. Here, we investigated a new EGFR-based combination therapy using the natural compound deguelin, showing that the *PIK3CA* mutation is associated with sensitivity to EGFR TKIs, and that deguelin has a combination effect with an EGFR TKI for the induction of apoptosis in HNSCC cells. Our data suggest that EGFR inhibitor–based therapy combined with deguelin showed a better benefit for HNSCC patients with the *PIK3CA* mutation, which are often resistant to EGFR TKI monotherapy. Therefore, the mutation of *PIK3CA* should be determined as a biomarker to administer deguelin for better clinical outcomes in cancer patients.

In this study, we indicated that deguelin at 1 μM reduced not only p-AKT expression, but also p-ERK expression. Previously, we reported that deguelin at 1 or 10 μM induced apoptosis [21] in HSC-4 cells, which were not sensitive to AG1478. Indeed, in this study, the viable cell number was almost the same between the combination of deguelin at 1 μM and AG1478 at 1 μM, and deguelin at 1 μM alone; however, this combination significantly reduced the viable cell number relative to AG1478 alone at 1 μM (data not shown). Taken together, this fact suggested that a high-dose deguelin monotherapy could be used for *PIK3CA*-mutated HNSCC patients, or the combination of low-dose deguelin and EGFR TKI could be used for *PIK3CA*-mutated HNSCC patients.

In HNSCC, *EGFR* and *KRAS* mutations seem to be rare [12,28]; however, mutation of PIK3CA occurs in 9%–11% of patients [13,14] and has been shown to activate the AKT signaling pathway [14]. In this study, we aimed to elucidate the importance of the EGFR signaling pathway in HNSCC using two kinds of cell lines with *EGFR* and *KRAS* wild-type genes with different gene statuses of *PIK3CA* [14,22].

Although *KRAS* mutation has been established as a potential biomarker for predicting the efficacy of erlotinib in lung cancer [11], little is known about a predictive marker for EGFR TKIs in HNSCC. Here, we clearly demonstrated that the *PIK3CA* mutation is associated with sensitivity to EGFR TKIs. Signal transduction through the PI3K/AKT pathway is mediated by the conversion of phosphatidylinositol 4,5-bisphosphate (PIP2) to phosphatidylinositol 3,4,5-triphosphate (PIP3) by PI3K. This reaction is antagonized by the phosphatase and tensin homolog deleted from chromosome ten (PTEN). In prostate cancer, PTEN expression is associated with EGFR inhibitor sensitivity [29]. On the contrary, PTEN expression in HNSCC was not associated with sensitivity to the EGFR inhibitor [15]. Therefore, further study was needed to determine the role of the PI3K signaling status in the efficacy of EGFR inhibitors in HNSCC.

## 4. Materials and Methods

### 4.1. Reagents

AG1478 and deguelin were purchased from Wako (Osaka, Japan). They were dissolved in dimethyl sulfoxide (DMSO) at a concentration of 50 mM stock solution, stored as small aliquots at −20 °C, and diluted with culture medium to make desired concentration (final DMSO concentration of less than 0.1%). Dulbecco’s modified Eagle medium (DMEM) was purchased from Nissui (Tokyo, Japan). FBS was from Hyclone (South Logan, UT, USA). Penicillin G potassium and streptomycin sulfate were from Meiji Seika (Tokyo, Japan). Detergent compatible (DC)™ Protein Assay Kit, based on the Lowry method, and avidin-conjugated horse radish peroxidase were from Bio-Rad (Richmond, CA, USA). N102 blocking reagent was from Nippon Oil and Fats (NOF) Corporation (Tokyo, Japan). Radio-Immunoprecipitation Assay (RIPA) Lysis Buffer System was purchased from and Santa Cruz Biotechnology (Dallas, TX, USA). Bovine serum albumin was from Sigma-Aldrich (St. Louis, MO, USA). Biotin-conjugated secondary antibody was from Biotin-conjugated goat anti-mouse IgG (H + L) (1:20,000) and biotin-conjugated goat anti-rabbit IgG (H + L) (1:20,000) was from Jackson ImmunoResearch (West Grove, PA, USA). Signals were detected with chemiluminescence reagent (Amersham, Buckinghamshire, UK). The chemiluminescence reagent “LuminataTM Forte Western HRP Substrate” was from Millipore (Billerica, MA, USA). Anti-glyceraldehyde 3-phosphate dehydrogenase (GAPDH) antibody (GT239, mouse monoclonal, 1:5000) was purchased from GeneTex (Irvine, CA, USA). Antibodies against phospho-AKT (Ser73) (D9E, rabbit monoclonal, 1:2000), total-AKT (rabbit polyclonal, 1:1000), total-p44/42 MAPK (Erk1/2) (137F5, rabbit monoclonal, 1:1000), PARP (rabbit polyclonal, 1:1000), and phospho-EGFR (Tyr1068) (D7A5, rabbit monoclonal, 1:1000) were purchased from Cell Signaling Technology (Tokyo, Japan). Antibodies against phospho-p44/42 MAPK (Erk1/2) (E-4, mouse monoclonal, 1:500) and total-EGFR (1005, rabbit polyclonal, 1:500) were purchased from Santa Cruz Biotechnology.

### 4.2. Cell and Cell Cultures

HSC-4 cells (JCRB0624) derived from tongue carcinoma were provided from the Human Science Research Resources Bank (HSRRB) (Osaka, Japan) and Ca9-22 cells (JCRB0625) derived from gingival carcinoma were from HSRRB through K. Hirose (Ohu University School of Dentistry, Koriyama, Japan) [30]. Their gene statuses are shown in Table 1, based on previous reports [14,22]. Cells were maintained in DMEM supplemented with 10% FBS and 100 units/mL penicillin G and 100 μg/mL streptomycin sulfate at 37 °C in a humidified atmosphere containing 5% CO_2_ and 95% air. Passage was done by tripsinization.

### 4.3. Cell Viability Assay

Ca9-22 and HSC-4 cells were seeded at density of 80 × 10^4^ cells/well in six-well tissue culture plates (Thermo Fisher Scientific, Hudson, NH, USA) and incubated in growth medium overnight, and further incubated with drugs. At the end of incubation, the cell numbers were determined using the trypan-blue dye exclusion method, as described elsewhere [21].

### 4.4. Protein Preparation and Western Blot Analysis

Cells were lysed by RIPA buffer and stored as small aliquots at −80 °C until use. Whole-cell extracts (20 μg/lane) was fractionated using sodium dodecylsuphate-polyacrylamide gel electrophoresis (SDS-PAGE). After electrophoresis, the proteins were electrotransferred onto polyvinylidene fluoride (PVDF) membranes, blocked with the blocking reagent NO1, and treated with the first antibody, and followed by the biotin-conjugated secondary antibody. Finally, signals were visualized with horse radish peroxidase-conjugated avidin and electrochemiluminescence reagent (Amersham, Buckinghamshire, UK). The signals detected by anti-GAPDH antibody, used as the loading control.

### 4.5. Vectors

Hemagglutinin (HA)-tagged E545K mutated p110α of human PI3K-cDNA expressing construct (pBABEpuro-HA-PIKCA-E545K1, HsCD00025919) was provided from the DNA Resource Core at Harvard Medical School (Boston, MA, USA). pAcGFP1-Hyg-C1 vector was purchased from Takara Bio USA (Mountain View, CA, USA).

### 4.6. Vector Construction and Transfection

To express mutant PI3K (E545K, constitutively active mutation) (PIK3CA G1633A) as a GFP-tagged protein, the PIK3CA mutant gene in pBABEpuro-HA-PIKCA-E545K1 was placed into pAcGFP1-Hyg-C1 vector using the In-Fusion™ cloning system according to manufacturer’s protocols. Briefly, the PIK3CA E545K insert without the HA region was amplified by PCR with the primer set: 5′-AAG CTT CGA ATT CTG ATG CCT CCA CGA CCA-3′ (forward) and 5′-CGG TAC CGT CGA CTG GTT CAA TGC ATG CT-3′ (reverse) and cloned into pAcGFP1-Hyg-C1, which has been linearized by PCR with the primer set: 5′-CAG TCG ACG GTA CCG CGG GC-3′ (forward) and 5′-CAG AAT TCG AAG CTT GAG CTC GAG ATC TGA-3′ (reverse) (underlined sequences were indicated for In-Fusion™ enzyme recognition tag sequences consisting of 15 mer). The mutant PIK3CA gene inserted in the pAcGFP1-Hyg-C1 vector was termed as pPIK3CA-GFP. The sequences were confirmed by DNA sequencing. The vector was transfected into cells with Xfect Transfection Reagent. The experimental protocols for the introduction of the vectors into *Escherichia. coli* (DH5α or JM109) or human cancer cells were approved by the ethical committee of Ohu University (Koriyama, Japan).

### 4.7. Protein Assay

The protein content in the lysates were measured using a DCTM Protein Assay Kit with bovine serum albumin as the standard.

### 4.8. Statistical Analysis

Statistical significance was calculated using Student’s *t*-test. *p*-values less than 0.05 were considered significant.

## 5. Conclusions

In conclusion, at first we demonstrated that the PIK3CA mutation is associated with sensitivity to EGFR TKIs. Second, we demonstrated the combination effect of AG1478 and deguelin in PIK3CA-mutated HNSCC. Therefore, our data in this study suggested that combination therapies using EGFR TKIs and deguelin are new therapeutic approaches to treat PIK3CA-mutated HNSCC.

## Figures and Tables

**Table 1:** *EGFR*, *KRAS*, and *PIK3CA* status of HNSCC cell lines.

Cell Lines	Origin	Gene Status *		
		*EGFR* (Exon 18, 19, 21)	*KRAS* (Exon 12, 13, 61)	*PIK3CA* (Exon 9, 20)
Ca9-22	gingiva	WT **	WT **	WT **
HSC-4	Tongue	WT **	WT **	G1633A (exon 9)

* Sheikh Ali et al. (2008) [22] and Kozaki et al. (2006) [14]. ** WT: wild type.
